# Suicide resilience, identity crisis and quality of life in burned adolescent**s**

**DOI:** 10.1002/nop2.1303

**Published:** 2022-08-08

**Authors:** Fateme Mohammadi, Khodayar Oshvandi, Seyed Reza Borzou, Masood Khodaveisi, Salman Khazaei, Mohammad Reza Shokouhi, Fatemeh Nouri, Mohammad Taheri, Majid Kalbasi

**Affiliations:** ^1^ Department of Pediatric Nursing, School of Nursing and Midwifery,Chronic Diseases(Home Care) Research Center and Autism Spectrum Disorders Research Center Hamadan University of Medical Sciences Hamadan Iran; ^2^ Department of Medical Surgical Nursing, School of Nursing and Midwifery, Chronic Diseases (Homecare) Research Center Hamadan University of Medical Sciences Hamadan Iran; ^3^ Department of Medical Surgical Nursing, School of Nursing and Midwifery, Chronic Diseases (Home Care) Research Center Hamadan University of Medical Sciences Hamadan Iran; ^4^ Department of community Health Nursing, School of Nursing and Midwifery, Mother and Child Care Research Center Hamadan University of Medical Sciences Hamadan Iran; ^5^ Research Center for Health Sciences Hamadan University of Medical Sciences Hamadan Iran; ^6^ Department of Medical Emergencies, School of Nursing and Midwifery Hamadan University of Medical Sciences Hamadan Iran; ^7^ Department of Pharmaceutical Biotechnology, School of Pharmacy Hamadan University of Medical Sciences Hamadan Iran; ^8^ Department of Microbiology, School of Medicine Hamadan University of Medical Sciences Hamadan Iran; ^9^ Plastic, Reconstructive and Aesthetic surgery fellowship Isfahan University of Medical Sciences Isfahan Iran

**Keywords:** adolescents, burn, identity crisis, quality of life, suicide resilience

## Abstract

**Aim:**

The aim of this study was to investigate the relationship between identity crisis and suicide resilience and quality of life in adolescents with burns in Iranian society.

**Design:**

A cross‐sectional study.

**Methods:**

Four hundred thirty adolescents with burn in three hospitals were selected via convenience sampling. They completed questionnaires online. Data were analysed by SPSS software version 22.

**Results:**

Findings showed a strong and inverse correlation between identity crisis with suicide resilience (*r* = −.92, *p* < .001) and quality of life (*r* = −.87, *p* < .001). Variables of suicide resilience, being a child of a divorced family, cause of burns, extent of burn, financial situation, gender and age can predict 68.74% of the variance in identity crisis in these patients.

**Patient or Public Contribution:**

Adolescents with burns suffer from an identity crisis, and although they report high resilience to suicide, they have a mediocre quality of life. Therefore, it is suggested that basic planning and extensive support be taken to improve physical and mental health, promote the quality of life and consequently reduce the identity crisis in these adolescents.

## 1 INTRODUCTION

Burns and their injuries are one of the most important health problems and one of the leading causes of death and disability in the world (Abdelbasset & Abdelhalim, 2020; Peck, 2012). Official statistics in Iran indicate 724,000 burn victims are hospitalized annually and majority of these patients are children and adolescents who are hospitalized due to self‐immolation or events (Khadem‐Rezaiyan et al., 2020). According to epidemiological studies in Iran, 53.9% of burns were due to hot liquids, 16.7% were due to hot objects, 15.4% were due to fire, 14.9% were due to explosions, 2.7% were due to electric burns, and 2.5% were related to chemical burn. This is while 41% of burns with the intention of self‐immolation and suicide have occurred mainly among adolescents and young people. Although many of these patients die of severe burns (Ahmadi, 2007; Mohammadhossini, Gheibizadeh, Saki Malehi, & Zarea, 2019), progress in the treatment and care of burn patients has increased their survival (Kazemzadeh, Rabiepoor, & Alizadeh, 2019).

However, burn severely affects all aspects of patients' lives. Clinical experiences of burn survivors indicate that this accident is associated with devastating stress (Haghi, 2018; Ilechukwu, 2002). Burning is associated with irreversible and chronic complications such as deformity, limb dysfunction, anxiety, sleep disturbances and nightmares (Batool, Malik, Fatima, & Manzoor, 2020; Haghi, 2018; Tehranineshat et al., 2020). Therefore, all burn patients experience psychological challenges and problems at different levels (Mohammadhossini et al., 2019). In this regard, burn patients state that quarrels and conflicts in life, curiosity of others, excessive family attention, economic problems, taunts, marriage prohibition, reduced communications, reduced social and professional activities are inseparable parts of their lives. It leads to isolation, avoiding communities, identity disorders, resilience and decreased quality of life (Kazemzadeh et al., 2019). Accordingly, paying attention to identity crises, resilience and the quality of life of burn patients are the most statistically significant responsibilities of the treatment team (Kornhaber, Bridgman, McLean, & Vandervord, 2016; Tehranineshat et al., 2020).

Meanwhile, adolescence is one of the most sensitive periods of human life because adolescence is transitional phase of growth and development between childhood and adulthood. The World Health Organization defines an adolescent as any person between the ages 12 and 19 (Hockenberry, Wilson, & Rodgers, 2021) experience many challenges and identity crisis during this period (Mohammadi, Rakhshan, Molazem, Zareh, & Gillespie, 2020). In Iran, although in recent years, respect for the rights of vulnerable groups, especially children, adolescents and women, have received a lot of attention, due to the traditional beliefs and attitudes of Iranian society, adolescents and especially female adolescents are strongly dominated by families. Thus, the choice of place of study, type of clothing and even their marriage is with the direct opinion of families. Sometimes, the opinion of adolescents is different from that of families, and this reinforces the background for differences, tensions and identity crises in adolescents (Kushendar & Fitri, 2018).

Identity crisis causes severe disruptions in adolescent personal adjustment, social relationships, academic achievement and career plan (Neculai, 2021). However, adolescent patients, especially those with burns in various organs, mainly the face, encounter many challenges, including physical and mental stress, depression and fear of dysfunction of organs, which leads to experiencing a more severe crisis in understanding their character and identity (Marwa & Tarimo, 2019; Mirlashari, Nasrabadi, & Amin, 2017). On the contrary, identity crisis due to the change in the organs and face of patients after burns may severely affect their resilience and causes patients to lose their tolerance and attempt suicide (Abtan, Naderifar, Rahnama, & Noorisanchooli, 2021; Mohammadhossini et al., 2019).

Resilience is one of the statistically significant concepts of positive psychology and refers to the dynamic process and positive adaptation to bitter and unpleasant experiences (Haghi, 2018; Tehranineshat et al., 2020). Improving resilience and increasing the threshold of tolerance of a person in the face of stressful conditions can moderate helplessness. It can also increase the morale, mental and physical health of individuals (Bazazian & Rajab, 2010). That is because resilience and its components such as self‐confidence, optimism, belief in individual abilities and acceptance act as barriers in stressful situations and increase human resilience and tolerance (Tehranineshat et al., 2020). Sometimes, therefore, it is necessary to evaluate the suicide resilience in these patients. On the contrary, resilience and optimism about the current situation can affect patients' quality of life (Kool, Geenen, Egberts, Wanders, & Van Loey, 2017).

Quality of life is an individual's attitudes and perceptions of their position towards the goals of expectations, their standards and priorities (Yazdi‐Ravandi et al., 2013). Quality of life is the difference between individuals' expectations and reality; therefore, the smaller the difference, the higher the quality of life (Abdelbasset & Abdelhalim, 2020; Spronk et al., 2020). In the present century, living a quality life is one of the main concerns of health professionals (Shojaei, 2008). However, studies show that resilience, self‐care, attitude towards life, spirituality and social support can tremendously affect the burn patients' quality of life (Abdelbasset & Abdelhalim, 2020; Spronk et al., 2020; Tehranineshat et al., 2020).

Although several studies have quantitatively and qualitatively examined the care challenges in burn patients and evaluated components such as resilience, self‐care and quality of life (Abdelbasset & Abdelhalim, 2020; Marwa & Tarimo, 2019; Mirlashari et al., 2017; Spronk et al., 2020; Tehranineshat et al., 2020) so far, no study has examined identity crisis, suicide resilience and the quality of life of adolescents with burns. Therefore, based on previously mentioned points, it was necessary to design and conduct a study to investigate the relationship between identity crisis, suicide resilience, and the quality of life in adolescents with burns in Iranian society. The three following aims were examined in the study “investigate identity crisis, suicide resilience and quality of life in burned adolescent's patients,” “investigate the relationship between identity crisis with suicide resilience and quality of life in adolescents with burns in Iranian society” and “identify predictor variables of identity crisis in burned adolescent's patients.”

## 2 METHODS

### 2.1 Study design and setting

This study is a cross‐sectional research from August to November 2021. This research was reported based on the Strengthening the Reporting of Observational Studies in Epidemiology (STROBE) statement, that is guidelines for reporting observational studies.

#### 2.1.1 Participants and sampling

In this study sample, sample size e has been estimated 355 samples based on study of Ahmed et al. with β = 80%, α = 0.05, effect size 0.35 and taking into account the 10% drop in each group (Waqas et al., 2016). Thus, patients who met the inclusion criteria were identified and invited to study. Therefore, the corresponding author contacted the office of nursing services and got the phone numbers of all the burned adolescent patients who were hospitalized in the burn wards of 3 hospitals affiliated with a university of medical sciences in the west and centre of Iran. Then, the corresponding author contacted these patients, explained the objectives of the study to them, and invited them to participate in the study. Finally, 355 burn patients who met the inclusion criteria were identified and invited to study. The inclusion criteria were as follows: being willing to participate, at least 12 months have passed since their illness and do not have other physical and mental disorders. The burned adolescent patients who failed to answer more than half of the items on their questionnaires or did not return their questionnaires were excluded. The participants were asked to complete and submit the questionnaires—a personal (demographic) characteristics questionnaire, identity crisis questionnaire, suicide resilience questionnaire and quality of life questionnaire—online. The researchers sent e‐mails and reminder messages to the patients, so that majority of the questionnaires (70.69%) were completely gathered in October. Three hundred four of the participants completed and returned the questionnaires via e‐mail or a social network. Thus, the response rate was 85.63%. The patients' reasons for not being participating in this study were that some of these patients were admitted for reconstructive surgery and were worried about the outcome of the surgery, or did not have the opportunity to fill out the questionnaires.

### 2.2 Questionnaire

#### 2.2.1 Demographic information questionnaire

In this study according to the World Health Organization definition of adolescence period, the age range of 12–18 years is considered as adolescence period. Also in the cultural context of Iranian society, those adolescents, especially female adolescents, may have married during this period. Therefore, examining demographic information is important. The questionnaire included age, gender, marital status, being child of divorce, cause of burns, extent of burn, financial condition and education.

##### Identity crisis questionnaire

The identity crisis questionnaire is a pencil–paper scale with 40 items. Ahmadi (1997) designed this questionnaire to assess the rate of suffering adolescents experience in an identity crisis. The emphasis in this questionnaire is on 10 dimensions and four questions which is graded according to the four‐point ordinal scale from without identity crisis (score 0) to a lot of identity crisis (score 3). This questionnaire includes 10 dimensions: 1—problem in following long‐term goals, 2—doubts in choosing a job, 3—not having a suitable model for friendship, 4—inappropriateness of sexual behaviour, 5—problems in recognizing and introducing religion, 6—not observing moral values, 7—non‐compliance with group commitment, 8—having a negative view of self, 9—lack of spirit of effort and initiative and 10—time management disorder. The maximum score in this questionnaire is 90, and the minimum is 0. The more a test taker's score approaches 90, the worst crisis he/she tends to undergo. The validity and reliability of the tool have been assessed. In the research by Dehshiri et al., the reliability of this questionnaire was calculated to be 83%, which represents a very acceptable validity coefficient (Dehshiri, 2005). The reliability of this questionnaire with Cronbach's alpha coefficient was estimated to be 0.91 in the present study.

##### Suicide resilience questionnaire

The suicide resilience questionnaire was first devised by Osman and et al., in 2004, to provide a multidimensional tool of suicide with applicability for different studies in adolescents and young adults. The scale is aimed to assess a person's perceived ability to deal with suicidal ideation, the availability of external resources and the person's assessment of their ability to deal with adverse events. This scale consists of 25 items that measure internal protective dimensions, external protective dimensions and emotional stability. This questionnaire is graded according to the six‐point Likert scale from strongly disagree (score 0) to strongly agree (score 5). Therefore, in this questionnaire, the test score will be between 0 and 125, and more scores indicate more resilience against suicide. The reliability of the whole questionnaire and scales has been reported ranging from 0.90 to 0.95. The validity of this tool is also calculated using different methods and all reported as acceptable (Madiyar & Nejati, 2016). In the present study, the face validity, content validity ratio (CVR) and content validity index (CVI) of this questionnaire were assessed by 20 experts (10 psychiatrists, 5 psychologists and 5 psychiatrists) for use in Iranian adolescents. All experts approved face validity and content validity (CVR = 0.57, CVI = 0. 83). In addition, the reliability of this questionnaire with Cronbach's alpha coefficient was estimated to be 0.93 in the present study.

##### Quality of life questionnaire

The Kidscreen Questionnaire is a self‐administered questionnaire for healthy children and adolescents with long‐term illness aged 8–18 years. The proxy copies are also available for parents or other caregivers. Kidscreen can be used in hospitals, medical centres and schools. Currently, there are three versions of Kidscreen available: 52, 27 and 10. The 27‐part version contains five dimensions: 1—Physical well‐being has five parts and measures the levels of physical activity, energy and fitness. 2—Psychological well‐being has seven parts and measures positive emotions, life satisfaction and moderate feelings. 3—It has seven items related to parents and adolescents' autonomy and measures the relationship with parents, the atmosphere in the home, sufficient age‐related freedom and the degree of satisfaction with financial resources. 4—Peer and social support has four items and examines the relationship with peers. 5—The school environment has four parts. It investigates the child and adolescent perception of cognitive capacity, learning, concentration and feelings about the school. This tool is designed on a five‐point Likert Scale (from 1 = never to 5 = always or from 1 = not at all to 5 = absolutely). The score is converted linearly to a scale of zero to 100 points, with 100 representing the best and zero representing the worst quality of life (Power et al., 2019; Ravens‐Sieberer et al., 2021). Nik Azin et al. evaluated the validity and reliability of this questionnaire in Iran and stated that this questionnaire has appropriate validity, and its reliability is estimated by Cronbach's alpha method for the whole instrument 74.8% (Nik‐Azin, Naeinian, & Shairi, 2013). The reliability of this questionnaire with Cronbach's alpha coefficient was estimated to be 0.89 in the present study.

### 2.3 Statistical methods

In this study, the collected data were analysed with SPSS software version 22. For this purpose, descriptive statistics (frequency, percentage, mean and standard deviation) were used. ANOVA and independent t‐test, respectively, were used to compare the mean score of identity crisis between three groups and two groups related to demographic variables. Pearson correlation coefficient was used to correlation between identity crisis, suicide resilience and quality of life. The significance level was considered *p* < .05. Then, the variables that had *p* < .25 were entered into the multiple linear regression model with a backward strategy. Backward strategy is a stepwise regression approach that begins with a full (saturated) model and at each step gradually eliminates variables from the regression model to find a reduced model that best explains the data. Hence, it is very effective for providing an accurate model. The researcher evaluated before performing multiple linear regression, hypotheses including normality of data, homogeneity of variance and independence of the residual.

### 2.4 Ethical considerations

The study design was approved by the ethics committee of the University of Medical Sciences (IR.UMSHA.REC.1400.385) and projects code (140009097533). At the beginning of study, the researcher introduced herself, explained the goals of the study and assured the participants that all information would remain confidential and they could withdraw from the study at any time. Finally, written informed consent was obtained from all the participants after providing them with sufficient information on the study.

## 3 RESULTS

### 3.1 Demographic information

Patients in this study were in the age ranging from 12 to 18 years with a mean age of 16.34 ± 1.53. Also, most of the patients were girls 181 (59.53%), high school's degree 139 (45.72%) and most of them were child of divorced family 181(59.53). Findings also showed a statistically significant relationship between identity crisis with being a child of a divorced family, cause of burns, extent of burn, financial situation, gender and age (Table 1).

**TABLE 1:** Participants' demographic characteristics and identity crisis score (number of participants = 304)

Demographic variables	*N* (%)	Moral courage Mean ± *SD*	*p*‐value
Age (year)			.044^a^
12–13	42 (13.81)	49.23 ± 1.65	
14‐15	121 (39.80)	53.98 ± 1.27	
16–18	141 (46.39)	59.23 ± 1.41	
Gender			.042^b^
Girl	181 (59.53)	63.86 ± 1.15	
Boy	123 (40.47)	58.23 ± 1.05	
Marital status			.381^b^
Single	297 (97.70)	57.34 ± 1.21	
Married	7 (2.30)	55.75 ± 1.34	
Education			.423^a^
Primary school	34 (11.18)	52.63 ± 1.53	
First high school	131 (43.10)	55.27 ± 1.76	
Secondary high school	139 (45.72)	56.87 ± 1.45	
Being child of divorce			.021^b^
Yes	181 (59.53)	61.12 ± 1.29	
No	123 (40.47)	55.27 ± 1.76	
Cause of burns			.027^a^
Events at working	104 (34.22)	47.62 ± 1.47	
Self‐immolation	105 (34.53)	62.31 ± 1.68	
Unexpected events	95 (31.25)	49.23 ± 1.65	
Extent of burn (per cent)			.028^a^
Less than 20	42 (13.81)	51.23 ± 1.65	
20–40	104 (34.22	57.27 ± 1.76	
40–60	139 (45.72)	59.87 ± 1.45	
More than 60	19 (6.25)	62.31 ± 1.68	
Financial situation ($/ month)			.033^a^
Less than $ 100	42 (13.81)	59.14 ± 1.32	
$ 100–300	181 (59.53)	52.27 ± 1.76	
More than $ 300	81 (26.64)	47.42 ± 1.47	

^a^ ANOVA test.

^b^ Independent *t*‐test.

### 3.2 Identity crisis, suicide resilience and quality of life in burned adolescent's patients

Patients participating in this study reported an identity crisis score of 62.05 ± 1.74, a suicide resilience score of 81.69 ± 1.31 and a quality of life care score of 52.17 ± 1.28 (Table 2).

**TABLE 2:** Means and standard deviations of the participants' identity crisis, quality of life and suicide resilience (number of participants = 304)

Variable	Dimensions	Mean ± *SD* (each dimension)	Mean ± *SD* (Total)	Median	Mode
Identity crisis	Problem with long‐term goals	69.98 ± 1.27	62.05 ± 1.74	51	49
	Doubts in choosing a job	67.98 ± 1.32			
	Not having a suitable model for friendship	61.86 ± 1.12			
	Inappropriateness of sexual behaviour	63.17 ± 1.24			
	Problems in recognizing and introducing religion	69.78 ± 1.27			
	Not observing moral values	68.23 ± 1.05			
	Non‐compliance with group commitment	73.86 ± 1.15			
	Having a negative view of yourself	70.98 ± 1.27			
	Lack of spirit of effort and initiative	68.23 ± 1.05			
	Time regulation disorder	69.65 ± 1.43			
Quality of life	Physical well‐being	52.31 ± 1.89	52.17 ± 1.28	39	34
	Psychological well‐being	51.63 ± 1.28			
	Communication with parents and autonomy of adolescents	52.41 ± 1.67			
	Social support and peers	51.87 ± 1.24			
	School environment	52.63 ± 1.44			
Suicide resilience	Internal protective	81.75 ± 1.32	81.69 ± 1.31	68	56
	External protective	88.27 ± 1.16			
	emotional stability	80.07 ± 1.64			

### 3.3 The relationship between identity crisis, suicide resilience and quality of life in burned adolescent patients

Findings in this study revealed a strong and inverse correlation between identity crisis with suicide resilience (*r* = −.92, *p* < .001) and quality of life (*r* = −.87, *p* < .001). Nevertheless, there is a strong and direct correlation between suicide resilience with quality of life (*r* = .88, *p* < .001) in burned adolescent patients. The direct and strong relationship between quality of life and resilience to suicide has a synergistic effect on reducing identity crisis, so improving the quality of life increases the resilience to suicide and consequently the synergistic effect of two variables is greatly reduced the identity crisis in these adolescents.

### 3.4 Predictors of identity crisis in burned adolescent's patients

The correlation matrix between variables showed in Table 3. The variables of suicide resilience, quality of life, being a child of a divorced family, cause of burns, extent of burn, financial situation, gender and age, which had a p‐value of smaller than 0.25, were entered into multiple linear regressions with the backward technique. Multicollinearity was checked using variance inflation factors (VIF). VIF of above 10 is sign of multicollinearity that can lead to inflation of variance and uncertainty in the resulting effect measures and VIF of 1 means that there is no multicollinearity among variables in the regression mode. Due to the collinearity statistic of between two variables suicide resilience (tolerance 0.35, VIF28.32) and quality of life (tolerance 0.31, VIF 31.02), the quality of life variable was removed from the model, so suicide resilience, being a child of a divorced family, cause of burns, extent of burn, financial situation, gender and age remained in the model and accounted for about 68.74% of the identity crisis variance in the burned adolescent patients (Table 4).

**TABLE 3:** Correlation matrix between variables (number of participants = 304)

Variables	Identity crisis	Suicide resilience	Quality of life	Being child of divorce	Cause of burns	Extent of burn	Financial situation	Gender	Age
Identity crisis	1								
Suicide resilience	0.87	1							
Quality of life	−0.92	0.88	1						
Being child of divorce	0.71	0.74	−0.81	1					
Cause of burns	0.68	0.69	0.71	0.63	1				
Extent of burn	0.72	0.74	−0.82	0.64	0.66	1			
Financial situation	−0.71	−0.67	0.69	0.71	0.31	0.32	1		
Gender	0.69	0.70	0.63	0.47	0.39	0.27	0.11	1	
Age	0.67	0.69	0.62	0.41	0.53	0.41	0.08	0.05	1

**TABLE 4:** Predictor variables of identity crisis in burned adolescents(number of participants = 304)

Factors	Non‐standard coefficients		Standard coefficients	R	R2	T	*p*‐value	Collinearity statistic	
	B	Standard error	β					Tolerance	VIF
Suicide resilience	−0.697	1.89	−0.711	6.55	42.90	0.36	.001	.35	28.32
Being child of divorce								0.20	4.87
Yes	Ref	‐	‐	‐	‐	‐	‐		
No	0.389	1.43	0.473	2.11	4.45	.27	.021		
Cause of burns								0.17	4.28
Events at working	Ref	‐	‐	‐	‐	‐	‐		
Self‐immolation	0.329	1.31	0.448	2.08	4.32	.25	.027		
Unexpected events	0.297	1.47	0.347	1.98	4.31	.20	.025		
Extent of burn								0.13	3.89
Less than 20	Ref	‐	‐	‐	‐	‐	‐		
20–40	0.327	1.30	0.437	2.0 2	4.08	.25	.028		
40–60	0.324	1.27	0.429	2.07	4.03	.26	.026		
More than 60	0.318	1.23	0.429	2.09	4.01	.25	.027		
Financial situation								0.12	3.84
Less than $ 100	Ref	‐	‐	‐	‐	‐	‐		
$ 100–300	0.317	1.27	0.421	1.98	3.92	.24	.033		
More than $ 300	0.311	1.25	0.421	1.98	3.92	.25	.032		
Gender								0.10	3.81
Girl	Ref	‐	‐	‐	‐	‐	‐		
Boy	0.171	1.09	0.231	1.76	3.09	.15	.042		
Age								0.10	3.81
12–13	Ref	‐	‐	‐	‐	‐	‐		
14–15	0.278	1.23	0.386	1.48	2.19	.22	.044		
16–18	0.281	1.20	0.386	1.48	2.19	.23	.042		

Adjusted R2: 68.74%.

## 4 DISCUSSION

The findings of the present study showed that although adolescents with burns suffer from an identity crisis and live a life with mediocre quality, they proved to have a high level of suicide resilience. The findings of this study also revealed a statistically significant inverse correlation between identity crisis and quality of life and suicide resilience. Suicide resilience, the cause of burns, the extent of burns, being the child of divorced parents, economic status, gender and age explain and predict a high percentage of variance changes in an identity crisis.

Although several studies have examined the quality of life, suicide resilience and identity crisis in burn patients or adolescents with other illnesses, no studies have evaluated the problem, suicide resilience and quality of life in adolescents with burns. The author, therefore, used other articles to examine the quality of life, identity and suicide resilience in burn patients or adolescents.

The identity crisis score in adolescents with burns in this study was 62.05 ± 1.74, indicating that they are suffering from an identity crisis. Consistent with the findings of this study, other studies in different parts of the world and with the different sample than this study show that burn patients experience an identity crisis due to changes in the appearance and function of the burn organ and consequently changes in occupational, educational and social interactions (Jain, Khadilkar, & De Sousa, 2017; Li, Zhou, & Wang, 2017; Mohammadhossini et al., 2019; Tehranineshat et al., 2020). Mohammadi et al. in Iran also stated that burn patients face severe psychological attacks. They remarked that these attacks affect their personality and identity so that they undergo a devastating identity and social crisis (Mohammadhossini et al., 2019). Jain et al. reported that burns patients report high stress, depression and low self‐esteem. These issues affect their perceptions and attitudes towards themselves and lead to experiencing an identity crisis (Jain et al., 2017).

Suicide resilience scores in an adolescent with burn were reported to be 81.69 ± 1.3 above, and there was an inverse relationship between resilience and identity crisis and quality of life. Meanwhile, in Iran, Tehrani et al. stated that the resilience in burn patients is 94.94 ± 0.138, and their tolerance for understanding and adaptation to existing conditions is great. There is also a direct correlation between resilience and quality of life, which is consistent with the findings of the present study (Tehranineshat et al., 2020). Nevertheless, the higher resilience score in the investigation by Tehrani et al. may be due to the resilience assessment in adults with burns, and it is clear that adults have more resilience for understanding and enduring the hardships and tragedies of life than adolescents. On the contrary, the present study evaluated suicide resilience, while the above investigation measured resilience in the general sense, and only a few questions focused on suicide resilience. Some studies in different parts of the world such as Jang et al. and Bibi et al. in line with the present study but with the different sample than this study stated that patients with burns have relatively high resilience level and there is an inverse relationship between resilience and stress levels. They also indicated a direct relationship between self‐confidence, self‐efficacy and optimism (Bibi, Kalim, & Khalid, 2018; Jang, Park, Chong, & Sok, 2017). Also Abrams et al. stated that burn patients suffer a lot of pain. However, they indicated that hope for recovery, motivation for life, empathy of others, spirituality and relationship with God increases their threshold of tolerance. These can also enhance resilience in these adult patients (Abrams, Ratnapradipa, Tillewein, & Lloyd, 2018).

The quality of life score of the participants in this study was estimated to be 52.17 ± 1.28, which indicates that the quality of life for adolescents with burns is moderate. There was also a direct relationship between the quality of life and the resilience of these adolescents. Consistent with the findings of this study, several studies in recent years have also examined the quality of life of patients with burns (Deeter et al., 2019; Gojowy, Kauke, Ohmann, Homann, & Mannil, 2019; Spronk et al., 2018; Tehranineshat et al., 2020).

In this regard, Tehrani et al. in Iran, but in the different sample than this study, described the quality of life of burn patients as relatively good, which is directly related to resilience and self‐care (Tehranineshat et al., 2020). The possible reason for this difference could be a difference in the age group under study, because Tehrani et al. have studied the adult age group, and it is clear that adults are more stable compared with adolescents in terms of occupation, emotion, identity and resilience, subsequently affecting the attitude towards life and quality of life. Deeter et al. and Jafaryparva et al., in other parts of the world and with the different sample than this study, also reported that the quality of life of adults with burns is moderate and is strongly influenced by the extent and complications of burns, which is consistent with the present study (Deeter et al., 2019; Jafaryparvar, Adib, Ghanbari Khanghah, & Kazem Nezhad Leyli, 2018).

In other parts of the world, Brewin et al. also stated that adult burn patients experience high levels of psychological stress that severely affect their quality of life; hence, they are not strong and they cannot continue living. Therefore, they need extensive support (Brewin & Homer, 2018). This similarity is probably because these patients experience changes in appearance, physical and social function following burns, which affect their interactions, self‐confidence, tolerance and quality of life (Spronk et al., 2020; Tehranineshat et al., 2020).

On the contrary, the findings of this study showed that 68.74% of the variance changes in an identity crisis in adolescents with burns are affected by the suicide resilience, cause of the burn, the extent of the burn, being a child of divorced parents, economic status, gender and age. There was not a study examining the predictors of identity crisis in adolescents with burns available to the researcher, so other studies that explained and predicted other pivotal variables were discussed. In this regard, in line with the findings of the present study, Li et al. using the meta‐analysis approach stated that the age, sex and that percentage of burns strongly affect the resilience of these patients consequently affecting the quality of life and understanding of their personality and identity (Li et al., 2017). Bibi et al. also stated that the resilience in burn patients is affected by their age, sex, economic status and stress level. Younger individuals, women and patients with low financial positions reported more stress and less resilience and experienced a more severe identity and social crisis accordingly (Bibi et al., 2018). Jafari Parva et al. stated that the quality of life of burn patients depends on the extent of the burn, consequences and complications of the burn dimension (Jafaryparvar et al., 2018). These similarities are probably because all of these studies have examined burn patients. Moreover, undoubtedly, the understanding of the disease that affects resilience, quality of life and identity crisis subsequently in these patients depends on the extent of the burn, the consequences, the well‐being and the support these patients receive.

Finally, it can be stated that according to the findings of this study, adolescents with burns, although they have reported high resilience to suicide, have a moderate quality of life and suffer from an identity crisis. For nurses who caring of adolescents with burns, It is very important that properly identify their problems and needs, and subsequently to reduce the occurrence of severe identity crises and to attempt re‐suicide in them, so the knowledge of these nurses about the relationship between identity crisis with quality of life and suicidal ideation as well as knowledge of the factors affecting the identity crisis in adolescents with burns can be very effective in improving their clinical performance. Accordingly, it is necessary to eliminate the initial planning and extensive support to improve physical and mental health, improve the quality of life and consequently reduce the identity crisis in these adolescents.

## 5 LIMITATIONS

One of the most statistically significant limitations of the present study was the non‐return of questionnaires. It was probably due to weakness, fatigue and lethargy in these patients. Accordingly, it is suggested to evaluate the identity crisis, resilience and quality of life of adolescents with burns in different communities with a larger sample size to obtain a more accurate estimation of the identity crisis in these patients.

## 6 CONCLUSIONS

Adolescents with burns experienced an identity crisis, which greatly affects their functionality, quality of life and resilience. On the contrary, suicide resilience, cause of the burn, the extent of the burn, being a child of divorced parents, economic status, gender and age were the elements resulting in the incidence of identity crisis in these adolescents, so that 68.74% % of the variance of their identity crisis was predictable.

## AUTHORS' CONTRIBUTIONS

FM, MK, KHO, SRB, MKH and MSH: Conception of the study, study design and data collection. FM and SKH: Data analysis. FM, MK, KHO, SRB, MKH, FN, MT and MSH: Primary manuscript draft and final manuscript revision and approval.

## CONFLICT OF INTEREST

The authors declare that they have no competing interests.

## ETHICS APPROVAL AND CONSENT TO PARTICIPATE

The institutional review board of the Hamadan University of Medical Science located in the west of Iran provided ethics approval (approval number: 1400.385). Also at the beginning of each interview, the researcher introduced herself and explained the goals of the study and assured that all information would remain confidential and that they could withdraw from the study at any time. The researchers provided the opportunity for participants to inform the researcher about their withdrawal from the study at any stage of the research and assured. Finally, written consent was obtained from study participants.

## CONSENT FOR PUBLICATION

Not applicable.

## Data Availability

The datasets used and/or analysed during the current study are available from the corresponding author on reasonable request.

## References

[nop21303-bib-0001] Abdelbasset, W. K. , & Abdelhalim, N. M. (2020). Assessing the effects of 6 weeks of intermittent aerobic exercise on aerobic capacity, muscle fatigability, and quality of life in diabetic burned patients: Randomized control study. Burns, 46(5), 1193–1200. 31911074 10.1016/j.burns.2019.12.013

[nop21303-bib-0002] Abrams, T. E. , Ratnapradipa, D. , Tillewein, H. , & Lloyd, A. A. (2018). Resiliency in burn recovery: a qualitative analysis. Social Work in Health Care, 57(9), 774–793. 30124390 10.1080/00981389.2018.1503213

[nop21303-bib-0003] Abtan, A. A. , Naderifar, M. , Rahnama, M. , & Noorisanchooli, H. (2021). Family experiences about burn patients: A content analysis study. Journal of Nursing Explorations, 1(1), 15–19.

[nop21303-bib-0004] Ahmadi, A. (2007). Suicide by self‐immolation: Comprehensive overview, experiences and suggestions. JBCR, 28(1), 30–41. 17211198 10.1097/BCR.0b013E31802C8878

[nop21303-bib-0005] Batool, I. , Malik, G. , Fatima, S. , & Manzoor, I. (2020). Investigation of dispositional optimism, psychological resilience, coping strategies and quality of life among burn survivors. Khyber Medical University Journal, 12(4), 310–314.

[nop21303-bib-0006] Bazazian, S. , & Rajab, A. (2010). Assessing the psychometric properties of the quality of life scale for diabetics (developing over aged 20‐60). Developmental Psychology, 6(24), 317–328.

[nop21303-bib-0007] Bibi, A. , Kalim, S. , & Khalid, M. A. (2018). Post‐traumatic stress disorder and resilience among adult burn patients in Pakistan: A cross‐sectional study. Burns and Trauma, 6, 1–6. 30009193 10.1186/s41038-018-0110-7 PMC6040608

[nop21303-bib-0008] Brewin, M. , & Homer, S. (2018). The lived experience and quality of life with burn scarring—The results from a large‐scale online survey. Burns, 44(7), 1801–1810. 30072198 10.1016/j.burns.2018.04.007

[nop21303-bib-0009] Deeter, L. , Seaton, M. , Carrougher, G. J. , McMullen, K. , Mandell, S. P. , Amtmann, D. , & Gibran, N. S. (2019). Hospital‐acquired complications alter quality of life in adult burn survivors: Report from a burn model system. Burns, 45(1), 42–47. 30477817 10.1016/j.burns.2018.10.010

[nop21303-bib-0010] Dehshiri, G. (2005). Investigating the relationship between religiosity and identity crisis among high school students in Yazd. Training and Education, 2(21), 78–98.

[nop21303-bib-0011] Gojowy, D. , Kauke, M. , Ohmann, T. , Homann, H.‐H. , & Mannil, L. (2019). Early and late‐recorded predictors of health‐related quality of life of burn patients on long‐term follow‐up. Burns, 45(6), 1300–1310. 31176508 10.1016/j.burns.2019.03.016

[nop21303-bib-0012] Haghi, S. (2018). The effect of resilience training on anxiety of patients with deformity due to burning injuries. International Journal of Healthcare, 20(3), 196–206.

[nop21303-bib-0013] Hockenberry, M. J. , Wilson, D. , & Rodgers, C. C. (2021). Wong's essentials of pediatric nursing‐e‐book. Elsevier health sciences.

[nop21303-bib-0014] Ilechukwu, S. T. (2002). Psychiatry of the medically ill in the burn unit. Psychiatric Clinics, 25(1), 129–147. 11912936 10.1016/s0193-953x(03)00055-8

[nop21303-bib-0015] Jafaryparvar, Z. , Adib, M. , Ghanbari Khanghah, A. , & Kazem Nezhad Leyli, E. (2018). Quality of life and associated factors in patients suffering from burns. Journal of Holistic Nursing And Midwifery, 28(3), 179–184.

[nop21303-bib-0016] Jain, M. , Khadilkar, N. , & De Sousa, A. (2017). Burn‐related factors affecting anxiety, depression and self‐esteem in burn patients: An exploratory study. Annals of Burns and Fire Disasters, 30(1), 30. 28592931 PMC5446905

[nop21303-bib-0017] Jang, M. H. , Park, J. , Chong, M. K. , & Sok, S. R. (2017). Factors influencing resilience of burn patients in South Korea. Journal of Nursing Scholarship, 49(5), 478–486. 28561890 10.1111/jnu.12311

[nop21303-bib-0018] Kazemzadeh, J. , Rabiepoor, S. , & Alizadeh, S. (2019). The quality of life in women with burns in Iran. World journal of plastic surgery, 8(1), 33–42. 30873360 10.29252/wjps.8.1.33. PMC6409149

[nop21303-bib-0019] Khadem‐Rezaiyan, M. , Aghajani, H. , Ahmadabadi, A. , Zanganeh, M. , Tavousi, S. H. , Sedaghat, A. , & Hasanabadi, S. E. (2020). Epidemiology of severe burns in north‐east of Iran: How is the burn size different in a developing country from developed ones? Burns Open, 4(1), 4–9.

[nop21303-bib-0020] Kool, M. B. , Geenen, R. , Egberts, M. R. , Wanders, H. , & Van Loey, N. E. (2017). Patients' perspectives on quality of life after burn. Burns, 43(4), 747–756. 28069345 10.1016/j.burns.2016.11.016

[nop21303-bib-0021] Kornhaber, R. , Bridgman, H. , McLean, L. , & Vandervord, J. (2016). The role of resilience in the recovery of the burn‐injured patient: An integrative review. Journal of Wound Care, 3, 41–50.

[nop21303-bib-0022] Kushendar, K. , & Fitri, H. U. (2018). The personal characteristics of an Islamic counselor in understanding identity crisis for adolescents. Islamic Guidance and Counseling Journal, 1(1), 17–24.

[nop21303-bib-0023] Li, J. , Zhou, L. , & Wang, Y. (2017). The effects of music intervention on burn patients during treatment procedures: A systematic review and meta‐analysis of randomized controlled trials. BMC Complementary and Alternative Medicine, 17(1), 1–14. 28302117 10.1186/s12906-017-1669-4 PMC5356403

[nop21303-bib-0024] Madiyar, M. , & Nejati, S. F. (2016). Validation of the suicide resilience inventory. Health Psychology, 4(4), 97–108.

[nop21303-bib-0025] Marwa, N. P. , & Tarimo, E. A. (2019). Provision of care to hospitalized pediatric burn patients: A qualitative study among nurses at Muhimbili National Hospital, Dar Es Salaam, Tanzania. BMC Nursing, 18(1), 1–10. 30911285 10.1186/s12912-019-0335-1 PMC6417030

[nop21303-bib-0026] Mirlashari, J. , Nasrabadi, A. N. , & Amin, P. M. (2017). Living with burn scars caused by self‐immolation among women in Iraqi Kurdistan: A qualitative study. Burns, 43(2), 417–423. 28341263 10.1016/j.burns.2016.08.019

[nop21303-bib-0027] Mohammadhossini, S. , Gheibizadeh, M. , Saki Malehi, A. , & Zarea, K. (2019). Burn patients' need for human caring: Content analysis study. Armaghane danesh, 24(3), 358–372.

[nop21303-bib-0028] Mohammadi, F. , Rakhshan, M. , Molazem, Z. , Zareh, N. , & Gillespie, M. (2020). Development of parental competence scale in parents of children with autism. Journal of Pediatric Nursing, 50, e77–e84. 31006517 10.1016/j.pedn.2019.04.006

[nop21303-bib-0029] Neculai, L. (2021). Case study regarding the identity crisis. New Trends in Psychology, 3(2), 10–28.

[nop21303-bib-0030] Nik‐Azin, A. , Naeinian, M. R. , & Shairi, M. R. (2013). Validity and reliability of health related quality of life questionnaire “KIDSCREEN‐27” in a sample of Iranian students. Iranian Journal of Psychiatry and Clinical Psychology, 8(4), 310–321.

[nop21303-bib-0031] Peck, M. D. (2012). Epidemiology and prevention of burns throughout the world. In Handbook of burns (pp. 19–60). Springer.

[nop21303-bib-0032] Power, R. , Akhter, R. , Muhit, M. , Wadud, S. , Heanoy, E. , Karim, T. , … Khandaker, G. (2019). Cross‐cultural validation of the Bengali version KIDSCREEN‐27 quality of life questionnaire. BMC Pediatrics, 19(1), 1–10. 30646887 10.1186/s12887-018-1373-7 PMC6334442

[nop21303-bib-0033] Ravens‐Sieberer, U. , Kaman, A. , Erhart, M. , Otto, C. , Devine, J. , Löffler, C. , … Siegel, N. A. (2021). Quality of life and mental health in children and adolescents during the first year of the COVID‐19 pandemic: Results of a two‐wave nationwide population‐based study. European Child and Adolescent Psychiatry, 19(9), 5220. 10.1007/s00787-021-01889-1 PMC8506100 34636964

[nop21303-bib-0034] Shojaei, F. (2008). Quality of life in patients with heart failure. HAYAT, 14(2), 5–13.

[nop21303-bib-0035] Spronk, I. , Legemate, C. , Oen, I. , van Loey, N. , Polinder, S. , & van Baar, M. (2018). Health related quality of life in adults after burn injuries: A systematic review. PLoS One, 13(5), e0197507. 29795616 10.1371/journal.pone.0197507 PMC5967732

[nop21303-bib-0036] Spronk, I. , Van Loey, N. E. , Sewalt, C. , Nieboer, D. , Renneberg, B. , Moi, A. L. , … Polinder, S. (2020). Recovery of health‐related quality of life after burn injuries: An individual participant data meta‐analysis. PLoS One, 15(1), e0226653. 31923272 10.1371/journal.pone.0226653 PMC6953837

[nop21303-bib-0037] Tehranineshat, B. , Mohammadi, F. , Tazangi, R. M. , Sohrabpour, M. , Parviniannasab, A. M. , & Bijani, M. (2020). A study of the relationship among burned patients' resilience and self‐efficacy and their quality of life. Patient Preference and Adherence, 14, 1361–1369. 32801666 10.2147/PPA.S262571 PMC7414971

[nop21303-bib-0038] Waqas, A. , Naveed, S. , Bhuiyan, M. M. , Usman, J. , Inam‐ul‐Haq, A. , & Cheema, S. S. (2016). Social support and resilience among patients with burn injury in Lahore, Pakistan. Cureus, 8(11), e867. 28070467 10.7759/cureus.867 PMC5218893

[nop21303-bib-0039] Yazdi‐Ravandi, S. , Taslimi, Z. , Saberi, H. , Shams, J. , Osanlo, S. , Nori, G. , & Haghparast, A. (2013). The role of resilience and age on quality of life in patients with pain disorders. BCN, 4(1), 24–30. 25337324 PMC4202549

